# Neighborhood socioeconomic disadvantage and school stock inhaler utilization in a statewide program

**DOI:** 10.3389/fpubh.2026.1750112

**Published:** 2026-02-09

**Authors:** Summer M. Reyes, Alexandra Knitter, Wen Wan, Lynn B. Gerald, Paige L. Hardy, Andrea A. Pappalardo, Erica Salem, Sam Weigley, Anna Volerman

**Affiliations:** 1 University of Chicago, Chicago, IL, United States; 2 University of Illinois at Chicago, Chicago, IL, United States; 3 Respiratory Health Association, Chicago, IL, United States; 4 Asthma and Allergy Foundation of America - MidStates Chapter, St. Louis, MO, United States

**Keywords:** Area Deprivation Index, asthma, child, pediatric, quick-relief inhaler, respiratory

## Abstract

**Background:**

The burden of asthma is disproportionately experienced by children from marginalized populations, including those from socioeconomically disadvantaged neighborhoods. Children can benefit from support beyond the home to counter negative asthma outcomes. Schools can stock quick-acting inhalers to ensure access to medication for any individual experiencing respiratory distress.

**Objectives:**

This study evaluated the relationship between stock inhaler use within schools and socioeconomic neighborhood disadvantage of schools.

**Methods:**

An observational study examined stock inhaler events at schools utilizing data from the Illinois Schools Undesignated Albuterol Program in the 2023–2024 school year. Schools were assigned Area Deprivation Index (ADI) rankings based on the average ADIs of the school's census block groups and categorized into quartiles. Simple and adjusted zero-inflated mixed-effects beta regressions were conducted to assess the relationship between school ADI quartile and stock inhaler use. Covariates included school type, rurality, and proportion of non-Hispanic White students.

**Results:**

Of 2,171 enrolled schools, 279 reported a total of 675 stock inhaler events. Most events involved students (*n* = 656). Individuals who used stock inhalers were 47% female, 38% Black, and 29% White. Schools in high disadvantage areas (ADI Q4) had higher stock inhaler event rates than schools in very low disadvantage areas (Q1) (OR = 1.49, 95% CI = 1.16, 1.90, *p* = 0.002). This relationship remained significant after adjusting for school and student characteristics and also was significant for moderate disadvantage (Q3) schools (Q3: OR = 1.30, 95% CI = 1.03, 1.66, *p* = 0.03; Q4: OR = 1.53, 95% CI = 1.20, 1.96, *p* < 0.001). In adjusted models, non-elementary schools had a lower stock inhaler event rate than elementary schools (OR = 0.67, 95% CI = 0.55, 0.81, *p* < 0.001), while the proportion of White students and rural location were not associated with event rates.

**Conclusions:**

We found that school neighborhood socioeconomic disadvantage was associated with stock inhaler utilization in Illinois schools. Future research is needed to investigate the driving factors, as stock inhaler programs can help address disparities in access to asthma care among children.

## Introduction

Asthma affects 4.3 million US school-aged children, and nearly 40% have uncontrolled disease, resulting in impaired quality of life, higher health care utilization, and decreased school attendance (1–3). The burden of asthma is disproportionately experienced by children of color and those from lower socioeconomic statuses (SES), with two to three times higher asthma prevalence in non-Hispanic Black and Puerto Rican children (4) as well as seven times higher mortality among non-Hispanic Black children compared to non-Hispanic White children (1). These disparities in asthma prevalence and outcomes persist due to various individual, systemic, and environmental factors, including the neighborhoods in which children live and participate (4).

Built environments, including neighborhoods, play a critical role in health, especially for individuals with asthma (5, 6). Asthma prevalence is higher in lower-income, urban communities than in their high-income counterparts (2). Socioeconomically disadvantaged and racially segregated neighborhoods, where Black and Hispanic children disproportionately live, are associated with many health risks, such as higher air pollution and poorer housing, that may contribute to disparities in asthma prevalence and outcomes (2). Understanding a neighborhood's socioeconomic conditions can be paramount to predicting asthma outcomes for its residents, including children.

One measure of a neighborhood's socioeconomic conditions is the Area Deprivation Index (ADI), a well-reported, validated tool used to understand neighborhood socioeconomic factors and their relationship to various health conditions, including asthma. Research shows that children with asthma from neighborhoods with higher ADI rankings (higher neighborhood disadvantage) had longer hospital lengths of stay, higher hospitalization costs, and higher readmission rates for asthma-related hospitalizations (7). Given their greater risk of asthma morbidity, children from neighborhoods with higher ADI ranking may benefit from additional support for their asthma management to counter these outcomes.

Schools are well-positioned to support asthma management as outlined in national guidelines (8, 9). However, schools face various barriers to effective implementation of these guidelines, including limited access to quick-relief medications and asthma care plans in schools (10–12). Stock inhalers have been proposed as an innovative and practical solution to address these challenges by increasing access to potentially life-saving medication for all individuals in schools (13). A stock inhaler is a quick-relief inhaler (commonly albuterol) that can be used by any individual to treat or prevent respiratory symptoms, thus providing an easy-to-use and cost-effective approach (14).

Over the past decade, 25 states, including Illinois, have passed legislation permitting schools to stock medications that provide quick relief for respiratory symptoms (e.g., albuterol) (15). Data is emerging about the impact of stock inhaler programs in schools. A study of a county-wide school stock inhaler program in Arizona found that 84% of students were able to return to class after using the stock inhaler, thereby preventing the loss of learning time that would have occurred if they had been sent home or transported to a hospital (16). Reports from two state-level programs describe similar results, with a majority of students who received the stock medication returning to class in Missouri (92%) and Texas (68%) (12, 17). These findings suggest positive outcomes for students; however, more evidence is needed about stock inhaler programs to determine whether they effectively reach neighborhoods and students disproportionately affected by asthma. Within Arizona's county-wide stock inhaler program, a study of stock inhaler utilization and neighborhoods showed that students in the second-most disadvantaged neighborhoods, as defined by ADI rankings, were the most frequent users of stock inhalers (18). However, it is unknown whether these findings demonstrating stock inhaler programs reach more disadvantaged populations would be replicated in different geographies (outside of Arizona) and larger-scale programs (state-level).

To fill this gap, this study aimed to describe the relationship between socioeconomic neighborhood disadvantage and stock inhaler use in a statewide stock inhaler program in Illinois, which in 2021 became the first state to allocate funding to implement such a program in its schools. Illinois faces similar challenges to the rest of the US, with only one in four children having good control of their asthma (12). Moreover, asthma disparities are evident in Illinois, with non-Hispanic Black children experiencing 5.5 times higher rates of asthma-related emergency department visits (12). In this study of Illinois' stock inhaler program, we hypothesized that those schools in high neighborhood socioeconomic disadvantage areas (high ADI rankings) would have a higher stock inhaler event rate compared to schools in very low disadvantage areas.

## Materials and methods

### Study design

This retrospective, observational study examined the stock inhaler events among school-aged children utilizing data from schools in the Illinois Schools Undesignated Albuterol (RESCUE) Program from the 2023–2024 school year (September 2023–June 2024). This study was deemed exempt by the University of Chicago Institutional Review Board.

### Program

In 2018, Illinois passed Public Act 100-0726, a law allowing schools to stock inhalers, and in 2021, it allocated funding for a statewide program. The Illinois RESCUE program was operated by the Asthma and Allergy Foundation of America – Midstates Chapter (AAFA) in 2023–2024. RESCUE aims to provide children in Illinois schools with access to stock inhalers, with a focus on communities disproportionately affected by health disparities. AAFA provided schools with medication, equipment, training, and an implementation handbook. Resources were distributed to schools using a classification system (Class 1–6) based on the size of a school's population and risk assignment determined by local data on pediatric asthma-related emergency department visits. Schools received quantities of each item based on their class, with each school receiving a minimum of three metered-dose inhalers, three spacers, and 10 disposable spacers.

### Data collection

We obtained data about schools participating in the RESCUE program and stock inhaler utilization events from AAFA. This data was collected between September 2023 and June 2024 using a tool developed based on the Illinois State Board of Education Undesignated Asthma Medication Reporting Form. Individual nurses or school staff registered in the RESCUE school database and were linked to their respective schools. Nurses reported incident-level information and characteristics of individuals who received albuterol (e.g., trigger, disposition). Individual stock inhaler event reports were aggregated to the school level. All school-level information (geolocation, enrollment, and student demographics) for the 2023–2024 academic year was derived from publicly available Illinois State Board of Education (ISBE) databases and linked to the RESCUE database (19). Data linkage was performed using multivariable fuzzy matching procedures with manual discrepancy review, resulting in matched school characteristics for 98% of events (school-level information could not be obtained for 13 events reported at 2 schools) (20).

**Figure 1:**
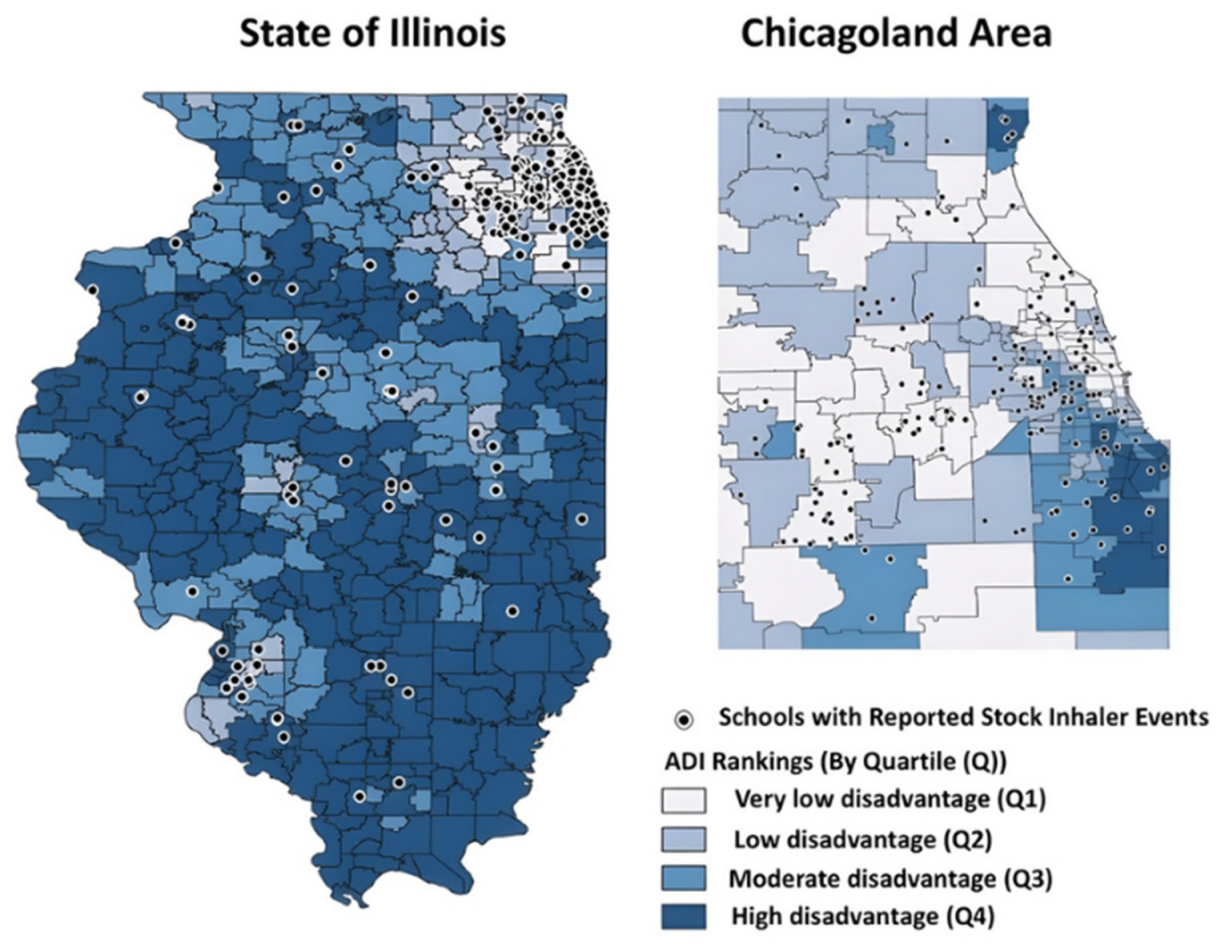
Maps of school socioeconomic disadvantage among Illinois schools with a stock inhaler event in 2023–2024 Illinois Schools Undesignated Albuterol Program.

Public datasets, in the form of shapefiles, from the US Census and the Chicago Data Portal were used for mapping and geocoding (21, 22). Geographic distribution of reported stock inhaler events and average school socioeconomic disadvantage was visualized using Illinois census block groups, Chicago Community areas, and Area Deprivation Index (Figure 1).

### Exposure

The 2022 Area Deprivation Index (ADI) census block group level rankings were used to calculate school disadvantage rankings in this study. The Area Deprivation Index is a measure created by the Health Resources & Services Administration (HRSA) and refined by the University of Wisconsin-Madison for the census block group level (23). It ranks socioeconomic disadvantage by census block, considering factors such as education, income, housing quality, and employment. Lower ADIs correspond to low neighborhood socioeconomic disadvantage, while higher ADIs correspond to high neighborhood socioeconomic disadvantage (23). The ADIs of schools reflect their catchment area, the geographic area surrounding the school where enrolled students could live. In alignment with the Lowe et al. study in Pima County, Arizona, we geographically defined schools' catchment areas by the boundaries of their corresponding school district's census block groups (18). As census block groups intersect school district boundaries, we used GIS spatial interpolation to connect census-level data with school district boundaries. This spatial interpolation accounted for the potential overlaps between the school geographies and provided the census-informed demographic information at the school district level. School catchment-level ADIs were calculated based on the average ADIs of the census block groups in the corresponding school district. Districts were then divided into quartiles based on their rankings: Quartile 1 (Q1) had very low disadvantage (range 1–3.2), Quartile 2 (Q2) had low disadvantage (range 3.3–4.9), Quartile 3 (Q3) had moderate disadvantage (range 5–7.1), and Quartile 4 (Q4) had high disadvantage (range 7.2–10). Individual schools were then assigned to the same quartile as their corresponding district. For schools in the Chicago Public Schools District, catchment areas and the subsequent ADI calculations and rankings were performed on the community area level rather than district wide.

### Outcome and covariates

The stock inhaler event rate was defined as the number of stock inhaler use events per total number of students in each school. School characteristics included rural location (as defined by the US Census), enrollment size, and school type (elementary, middle, or high school). School-level student characteristics included information on gender, race/ethnicity, low-income status, and use of Individualized Education Programs. ISBE school-level student characteristics with fewer than 10 students were treated as missing; the only exception was for race/ethnicity, which were set to zero and reassigned to an Unknown race/ethnicity category.

### Data analysis

Descriptive statistics summarized the characteristics of stock inhaler events, along with participating RESCUE schools and their students. Kruskal-Wallis, Chi-squared, and Fisher's exact tests evaluated differences in school characteristics (e.g., number of events, school type, school enrollment size, students' race/ethnicity, students' SES) by ADI quartile.

Unadjusted and adjusted zero-inflated mixed-effects beta regressions (via R package glmmTMB) were used to model stock inhaler event rates across the four ADI quartiles, accounting for within-county and within-district associations (three-level nested data structure) (24). Since most enrolled schools (87%) did not report any stock inhaler events, zero-inflated models were used, which allowed us to separately account for schools reporting zero vs. any events (binary model) while performing a primary analysis of events for those who reported at least one event (continuous model).

In the adjusted models, covariates included school type (binary, elementary vs. non-elementary), rurality (binary, rural vs. not rural), and proportion of non-Hispanic White students (continuous, range 0–1). Model covariates were selected based on the Akaike information criterion (AIC). Multicollinearity was assessed using variance inflation factor of ≥5). Schools were excluded from regression analyses if they had missing data for school enrollment (affecting event rate calculation) or any of the covariates.

Statistical analyses were performed using R Statistical Software (v.4.2.3; R Core Team 2023). QGIS 3.14.16 was used for geocoding and geospatial mapping.

## Results

A total of 2,171 schools received supplies as part of the RESCUE program in the 2023–2024 school year. The median enrollment of these schools was 406 students [interquartile range (IQR): 268–579] (Table 1). Of enrolled schools, 8.9% were rural and 67% were elementary schools (Table 1). Overall, the median student population of these schools was 49% female (IQR: 47%−51%) and 54% low-income (IQR: 30%−82%) (Table 2).

**Table 1:** Characteristics of schools that participated in the stock inhaler program in Illinois and reported stock inhaler events in 2023–2024, based on Area Deprivation Index (ADI) rankings.

**Characteristics**	**All participating schools**, ***n*** = **2171**^**a**^						**Schools reporting events**, ***n*** = **279**^**b**^					
	**Total** ***n*** = **2,171**	**Q1** ***n*** = **635**	**Q2** ***n*** = **541**	**Q3** ***n*** = **580**	**Q4** ***n*** = **415**	*p*−*value*^c^	**Total** ***n*** = **279**	**Q1** ***n*** = **97**	**Q2** ***n*** = **68**	**Q3** ***n*** = **65**	**Q4** ***n*** = **49**	*p*−*value*^d^
Schools with stock inhaler events, *n* (%)	279 (13%)	97 (15%)	68 (13%)	65 (11%)	49 (12%)	0.2	–	–	–	–	–	–
Student enrollment, median (IQR)	406 (268, 579)	497 (345, 670)	432 (301, 595)	372 (247, 529)	289 (197, 411)	< 0.001	502 (336, 766)	572 (426, 883)	530 (330, 779)	457 (329, 732)	334 (242, 525)	< 0.001
Rural, *n* (%)	193 (8.9%)	3 (0.5%)	10 (1.9%)	79 (14%)	101 (24%)	< 0.001	12 (4.3%)	0 (0%)	0 (0%)	5 (7.7%)	7 (14%)	< 0.001
Elementary school, *n* (%)	1,443 (67%)	426 (68%)	382 (71%)	381 (66%)	254 (62%)	0.03	187 (67%)	66 (68%)	48 (71%)	42 (65%)	31 (63%)	0.8

IQR, Interquartile range [represented as (Quartile 1, Quartile 3) of data]; Q, ADI Quartile based on catchment-level ADI rankings.

^a^Missing data among all participating schools: 28 schools for student enrollment, 3 schools for rurality, 16 schools for grade served (elementary school).

^b^Missing data among schools reporting events: 2 schools for student enrollment.

^c^Kruskal Wallis rank sum test; Pearson's Chi-squared test.

^d^Kruskal Wallis rank sum test; Pearson's Chi-squared test; Fisher's exact test.

**Table 2:** Characteristics of students within schools that participated in the statewide stock inhaler program and reported stock inhaler events in 2023–2024, by Area Deprivation Index (ADI) rankings.

**Student characteristics**	**All participating schools**, ***n*** = **2,171**^**a**^ **Median Proportion (IQR)**						**Schools reporting events**, ***n*** = **279**^**b**^ **Median Proportion (IQR)**					
	**Total** ***N*** = **2,171**	**Q1** ***N*** = **635**	**Q2** ***N*** = **541**	**Q3** ***N*** = **580**	**Q4** ***N*** = **415**	*p*−*value*^c^	**Total** ***N*** = **279**	**Q1** ***N*** = **97**	**Q2** ***N*** = **68**	**Q3** ***N*** = **65**	**Q4** ***N*** = **49**	***p-valu*** **e** ^c^
Female	0.49 (0.47, 0.51)	0.48 (0.47, 0.50)	0.48 (0.46, 0.50)	0.49 (0.47, 0.51)	0.49 (0.47, 0.51)	0.054	0.48 (0.47, 0.50)	0.48 (0.47, 0.50)	0.48 (0.47, 0.50)	0.49 (0.47, 0.50)	0.49 (0.47, 0.51)	0.4
**Race/Ethnicity**												
White	0.39 (0.03, 0.76)	0.51 (0.31, 0.70)	0.26 (0.03, 0.65)	0.11 (0.00, 0.84)	0.56 (0.02,0.91)	< 0.001	0.41 (0.11, 0.66)	0.51 (0.34, 0.66)	0.25 (0.05, 0.57)	0.24 (0.00, 0.54)	0.51 (0.21, 0.89)	< 0.001
Black	0.06 (0.00, 0.29)	0.04 (0.00, 0.10)	0.05 (0.00. 0.18)	0.12 (0.00, 0.81)	0.14 (0.00, 0.65)	< 0.001	0.08 (0.02, 0.20)	0.06 (0.02, 0.11)	0.05 (0.03, 0.13)	0.15 (0.02, 0.50)	0.18 (0.00, 0.53)	< 0.001
Hispanic	0.14 (0.04, 0.38)	0.18 (0.11, 0.33)	0.30 (0.11, 0.64)	0.08 (0.00, 0.43)	0.04 (0.00, 0.11)	< 0.001	0.17 (0.06, 0.38)	0.18 (0.11, 0.32)	0.37 (0.15, 0.75)	0.10 (0.04, 0.51)	0.05 (0.00, 0.12)	< 0.001
Low-income	0.54 (0.30, 0.82)	0.29 (0.16, 0.51)	0.54 (0.29, 0.79)	0.71 (0.43, 0.92)	0.65 (0.52, 0.87)	< 0.001	0.49 (0.29, 0.73)	0.30 (0.18, 0.46)	0.50 (0.27, 0.78)	0.62 (0.46, 0.89)	0.64 (0.53, 0.83)	< 0.001
With individualized education programs	0.15 (0.12, 0.19)	0.14 (0.12, 0.17)	0.15 (0.12, 0.18)	0.16 (0.13, 0.19)	0.18 (0.14, 0.22)	< 0.001	0.15 (0.13, 0.18)	0.15 (0.12, 0.17)	0.15 (0.13, 0.18)	0.16 (0.14, 0.19)	0.16 (0.13, 0.22)	0.002

IQR, Interquartile range, represented as (quartile 1, quartile 3); Q, ADI Quartile.

^a^Missing data among all participating schools: 38 schools for sex (female), 28 schools for race/ethnicity, 66 schools for low-income, 63 with Individualized Education Programs.

^b^Missing data among schools reporting events: 2 schools for sex (female), 2 schools for race/ethnicity, 7 schools for low-income, 4 with Individualized Education Programs.

^c^Kruskal Wallis rank sum test.

Among the 2,171 enrolled schools, 279 schools (12.85%) reported a total of 675 stock inhaler events during the 2023–2024 school year (Table 3). Students utilized the stock inhaler in 97% of events, while staff accounted for 3% of events. Among the 675 events, 47% of individuals were female, with a median age of 11 years old (IQR: 8–14). Thirty-eight percent of events occurred among non-Hispanic Black individuals, followed by 29% among non-Hispanic White individuals. In most events (76%), individuals returned to class after receiving treatment with a stock inhaler. In terms of events by ADI quartiles, Q1 schools had the largest proportion of total events while Q4 schools had the smallest proportion of total events (Q1: 34%, *n* = 229; Q2: 21%, *n* = 143; Q3: 27%, *n* = 180; Q4: 18%, *n* = 123); using expected counts proportional to the number of schools with events in each quartile, this difference was not statistically significant (*p* = 0.08).

**Table 3:** Characteristics of stock inhaler events in statewide program in Illinois in 2023–2024 school year, *n* = 675.

**Characteristic**	***N* (%)**
Age, median (IQR)	11.0 (8.0, 14.0)
Female	315 (47%)
**Race/ethnicity**	
Black	256 (38%)
White	194 (29%)
Hispanic or Latino	119 (18%)
Asian	30 (4.4%)
American Indian or Alaska Native	16 (2.4%)
More than one race	5 (0.7%)
**Disposition after event**	
Returned to class/responsibilities	514 (76%)
Left with parent/guardian or friend/family member	135 (20%)
Sent home	12 (1.8%)
Transported by emergency medical services	12 (1.8%)

IQR, Interquartile range, represented as (Quartile 1, Quartile 3).

Regarding school-level characteristics, for the 279 schools that reported events (Table 1), the median enrollment was 502 students (IQR: 336–766). Enrollment differed significantly by ADI quartile, with lower median enrollment for schools with higher ADI (*p* < 0.001). Rurality also differed significantly across ADI quartiles, with a greater proportion of rural schools in ADI quartiles Q3 and Q4 (*p* < 0.001). There was no significant difference in the proportion of elementary schools in each ADI quartile for the 279 schools with reported events (*p* = 0.4); however, for all 2,171 schools participating in the RESCUE program, the proportion of elementary schools differed significantly across ADI quartiles (*p* = 0.03).

In terms of student populations at the 279 schools reporting events (Table 2), the median proportion of students who were low-income differed across schools' ADI quartiles (*p* < 0.001), with a greater proportion of students who were low-income in the more disadvantaged quartiles (Q3 and Q4). Additionally, there was a significant relationship between ADI quartiles and racial/ethnic demographics of the schools reporting events (*p* < 0.001). Specifically, the median proportion of Black and Hispanic students differed between quartiles, with a greater proportion of Black students in schools in Q3 and Q4, as well as the greatest proportion of Hispanic students in schools in Q2 (both *p* < 0.001). The proportion of White students was greatest in schools in Q1 and Q4 (*p* < 0.001). There were no significant differences found for student gender between ADI quartiles among schools with events (*p* = 0.4).

When examining whether schools had stock inhaler events across ADI quartiles (Table 4), there were no differences in the odds of reporting zero events for Q2, Q3, or Q4 as compared to Q1. Non-rural schools were more likely to report any events than rural schools in adjusted models (*p* = 0.02).

**Table 4:** School stock inhaler event rates in statewide program for Illinois schools^a^, by school neighborhood disadvantage quartiles, unadjusted and adjusted models^b^.

**Variable**	**Unadjusted model**			**Adjusted model**		

	**Odds ratio**	**95% CI** ^c^	***p-value***	**Odds Ratio**	**95%CI** ^c^	***p-value***
**Binary model (0 events vs**. ≥**1 events)**^d^						
**ADI Quartiles** ^e^						
Q1 (Intercept)	—	—	—	—	—	—
Q2	1.21	0.76, 1.92	0.4	1.22	0.76, 1.96	0.4
Q3	1.29	0.82, 2.03	0.3	1.18	0.73, 1.88	0.5
Q4	1.40	0.83, 2.35	0.2	1.17	0.68, 2.00	0.6
**School characteristics**						
Rural	—	—	—	2.70	1.20, 6.08	0.02
Non-elementary^f^	—	—	—	0.99	0.73, 1.34	>0.9
**Student characteristics**						
Percent White	—	—	—	1.19	0.59, 2.40	0.6
Continuous model (event rate for ≥1 events)^g^
**ADI Quartiles** ^d^						
Q1 (Intercept)	—	—	—	—	—	—
Q2	1.02	0.80, 1.29	0.9	1.01	0.80, 1.28	0.9
Q3	1.26	0.999, 1.59	0.051	1.30	1.03, 1.65	0.03
Q4	1.49	1.16, 1.90	0.002	1.53	1.20, 1.96	< 0.001
**School characteristics**						
Rural	—	—	—	0.91	0.58, 1.41	0.7
Non-elementary^f^	—	—	—	0.66	0.55, 0.80	< 0.001
**Student characteristics**						
Percent White	—	—	—	1.03	0.76, 1.40	0.8

^a^39 of the 2,171 schools were excluded in regression analyses due to missing/censored data in model covariates (11 for school characteristics; 28 for student characteristics).

^b^Unadjusted and adjusted models account for random effects of schools within the same school district and/or county. Adjusted model includes covariates of school type (non-elementary vs. elementary grades), rurality (rural vs. not), and percent of non-Hispanic White students.

^c^CI, confidence intervals.

^d^Zero-inflated mixed effects beta regression results shown for the continuous event rate models, operationalized as the number of stock inhaler use events per total number of students in each school.

^e^Q, Quartile; higher quartile numbers reflect that the school has greater neighborhood disadvantage.

^f^ Non-elementary defined as middle and high schools.

^g^Zero-inflated mixed effects beta regression results shown for the binary event models, operationalized as having zero stock inhaler use events vs. having any events.

Among the 279 schools that reported at least one event (Table 4), schools in high disadvantage areas (Q4) had a 49% higher stock inhaler event rate than schools in very low neighborhood disadvantage areas (Q1) (OR = 1.49, 95% CI = 1.16, 1.90, *p* = 0.002). After adjusting for covariates, this trend persisted for Q4, corresponding to a 53% higher event rate compared to Q1 schools (OR = 1.53, 95% CI = 1.20, 1.96, *p* < 0.001). In the adjusted model, this trend also became statistically significant for Q3 schools with a 30% higher event rate compared to Q1 schools (OR = 1.30, 95% CI = 1.03, 1.66, *p* = 0.03). In the adjusted model, non-elementary schools (middle and high schools) had a 67% lower stock inhaler event rate compared to elementary schools (OR = 0.67, CI = 0.55, 0.81, *p* < 0.001). There were no significant associations between adjusted stock inhaler event rates and the proportion of non-Hispanic White students or the rurality of the school.

## Discussion

This observational study found that socioeconomic disadvantage was associated with stock inhaler utilization events in Illinois schools. To our knowledge, this is the first study to examine the neighborhood context in relation to a statewide stock inhaler program. In this study, very low disadvantage (Q1) schools most frequently reported stock inhaler use events. However, among schools reporting at least one event, the highest stock inhaler event rates were in highly disadvantaged area schools (Q4). Further, we discovered a relationship between school type and stock inhaler events, with elementary schools having higher stock inhaler event rates compared to non-elementary schools.

Our findings regarding school neighborhood disadvantage and its association with stock inhaler use complement prior findings in Pima County, Arizona, and expand on them by demonstrating similar results at a larger, statewide scale. Lowe et al. found that schools located in areas with the second highest level of disadvantage reported the most frequent use of stock inhalers (18). In contrast, our study ascertained that schools in the highest level of disadvantage had the highest rates of stock inhaler use. The differences may be due to the larger scale of our study at the state-level compared to a county-wide analysis of stock inhalers and neighborhoods. Additionally, Arizona and Illinois have different community makeups. Pima County, Arizona, had a population of 1.08 million in 2020, with 51% non-Hispanic White, 37% Hispanic, and 5% non-Hispanic Black individuals (25). On the other hand, the state of Illinois had a population of 12.7 million in 2020, 59% non-Hispanic White, 19% Hispanic, and 15% non-Hispanic Black individuals (25). In terms of rurality, 7.5% of the Pima County population lived in rural areas, while 11.5% of Illinois residents lived in rural areas (26). While these key differences in scale and population may have contributed to the variation in results, both studies highlight a correlation between higher neighborhood disadvantage and higher stock inhaler use among schools, suggesting that stock inhalers help ensure medication access for marginalized populations.

Although schools in high disadvantage neighborhoods (Q4) exhibited the highest rates of stock inhaler events, it is notable that very low disadvantage (Q1) schools made up the greatest proportion of stock inhaler use events among the quartiles, even after accounting for the number of schools. This finding may have occurred for several reasons. Very low disadvantage schools may have enrolled in the program earlier, as enrollment occurred on a rolling basis. For instance, many schools in Chicago were not a part of Q1 and enrolled later in the school year. Earlier enrollment would have provided Q1 schools with more opportunities to utilize stock inhalers over the school year. In addition, Q1 schools may have more students with documented asthma diagnoses and asthma action plans, potentially leading to more frequent stock inhaler use due to nurses feeling more comfortable with giving an inhaler to a child with documented asthma vs. a child without known asthma diagnosis.

This study also found that elementary schools utilized stock inhalers more than middle and high schools. This finding is consistent with asthma epidemiology, as children at 7–11 years tend to have more allergy- and exercise-induced exacerbations as well as more severe exacerbations due to weather changes and environmental factors (27). In comparison, by adolescence (12–18 years old), asthma remission is common, with rates from 16% to 60%, which may lead to lower asthma event rates and less quick-acting inhaler use for this age group (27). Further, adolescents experience different challenges with asthma than their younger counterparts, such as feeling different from others due to chronic illness and stigmatization from peers (28). These experiences among adolescents can often lead to under-utilization and non-adherence to asthma management, including potentially with stock inhalers, which can contribute to worsened disease control and more severe symptoms for this age group.

This research highlights the benefits of understanding the nuanced factors that influence the health of individuals with asthma, particularly when implementing policies like stock inhalers in schools. These study results can be used to further investigate the drivers of higher stock inhaler use in high neighborhood disadvantage schools (as represented by higher ADI). Factors associated with socioeconomically disadvantaged neighborhoods may play an important role. High socioeconomically disadvantaged areas, where there is substandard housing and greater poverty, were also often historically redlined (27). Research shows that residents in areas more likely to be redlined have a 2.4 times higher rate of asthma-related emergency visits than those in areas less likely to be redlined (4). Additional environmental factors, such as higher exposure to air and water contaminants, may contribute to more frequent asthma exacerbations for these students as compared to those in very low disadvantage school areas (4). It is possible that children may experience such harmful exposures not just at home but in spaces like schools, where they spend most of their time and also where 10%−25% of childhood injuries and emergencies already occur (28). Further, stock inhalers may fill a gap for these students who may have financial obstacles related to asthma management. Students in disadvantaged neighborhoods may also have higher uninsured or underinsured rates, or alternatively may be unable to afford a second inhaler to use in school (9). Altogether, future research examining the potential complex drivers of stock inhaler use is vital to help contextualize these study findings and better understand how stock inhaler programs can be most effective for students and communities.

Our study had several limitations. Schools were invited to participate in the RESCUE program and then they enrolled and reported events in alignment with state legislation and programmatic requirements. As such, the findings may be affected by selection bias, which could potentially overrepresent certain types of schools. For example, high disadvantage schools may experience difficulties with enrolling in the program or reporting events due to limited resources, including staff, time, and technology access. In addition, there was no universal start date for the RESCUE program in 2023–2024, as the initial supplies were distributed to schools in the first few months of the school year, and schools enrolled in the program on a rolling basis. Because schools had differing lengths of time in the program, monthly event rates could not be calculated and were not used as outcomes in analyses. Furthermore, schools may have underreported events, given that it was the first year of the program. Schools were required to report to both the state and RESCUE, which may have affected the consistency of reporting events to both. There were 1,900 reports of stock inhaler use reported to the state, and 675 events reported to the RESCUE. Some schools may have reported to the state only, some to RESCUE only, and some to both RESCUE and the state. Both rolling enrollment of schools and underreporting of events may have impacted the findings about event rates by ADI quartiles, given that community socioeconomic conditions, asthma prevalence, and other factors may contribute to over- or under-estimation of the association. Additionally, since data were reported at an event level rather than an individual level, our event rate likely includes students who used the stock inhaler multiple times during the year. More broadly, this research may not be generalizable to the rest of the country and may only be comparable to states with similar demographic characteristics to Illinois.

Additionally, study limitations may be related to the ADI rankings. The latest version of the ADI rankings available was based on 2018–2022 data. The ADI status of neighborhoods may have changed and may not accurately reflect the socioeconomic disadvantage of schools and their students in 2023–2024. However, the use of ADI quartiles considers this potential minimal change in ADI score. Second, for the dense/highly urban area of Chicago, we used the more granular community areas to calculate ADIs by individual schools (rather than the school-district defined census block group level used for other school districts); however, it may still not accurately reflect the socioeconomic differences of Chicago's school neighborhoods due to the density of Chicago. We believe this representation is the most accurate available for socioeconomic disadvantage differences within the city. Finally, ADIs were averaged at the community/district level and categorized into quartiles, which may have impacted the magnitude of the actual association between ADI quartiles and stock inhaler use, as it may mask within-district heterogeneity of neighborhood socioeconomic disadvantage. However, this approach aligns with prior studies using ADIs, which allows for comparison across studies.

Large-scale school stock inhaler programs help to ensure that all children, regardless of their neighborhood and background, have access to life-saving medication in case of respiratory distress. We show that schools' neighborhood socioeconomic disadvantage was associated with the use of stock inhalers in schools across the state of Illinois. Future research is needed to investigate the driving factors for these findings, which will help advocate for stock inhaler programs nationwide. Stock inhaler programs can be instrumental in increasing medication access in case of emergency, helping address asthma disparities, and ensuring all children have the chance to thrive.

## Data Availability

The original contributions presented in the study are included in the article/supplementary material, further inquiries can be directed to the corresponding author.
